# Treatment of brain metastases from non-small cell lung cancer: preclinical, clinical, and translational research

**DOI:** 10.3389/fonc.2024.1411432

**Published:** 2024-10-29

**Authors:** Parth J. Sampat, Alyssa Cortese, Alexandra Goodman, Ghanshyam H. Ghelani, Michael D. Mix, Stephen Graziano, Alina Basnet

**Affiliations:** ^1^ Division of Hematology and Medical Oncology, Department of Medicine, SUNY Upstate Medical University, Syracuse, NY, United States; ^2^ Department of Radiation Oncology, SUNY Upstate Medical University, Syracuse, NY, United States

**Keywords:** brain metastases, non-small cell lung cancer, non-small cell adenocarcinoma, lung cancer, squamous cell lung cancer

## Abstract

Lung cancer is the second most common type of cancer and is the leading cause of cancer-related deaths in the United States. Approximately 10-40% of patients with solid tumors develop brain metastases, with non-small cell lung cancer accounting for approximately 50% of all cases of patients with brain metastases. Many management options are available which can include surgery, radiation, and systemic therapy. A variety of factors go into the selection of management of brain metastases. In this review, we will focus on the treatment strategies and optimizing the management of brain metastases in patients with non-small cell lung cancer.

## Introduction

Lung cancer is the second most common type of cancer and is the leading cause of cancer-related deaths in the United States (1). Although the most recent World Health Organization (WHO) classification does not classify non-small cell lung cancer separately (2), historically, lung cancers can be divided into small cell lung cancer (SCLC), and non-small cell lung cancer (NSCLC). NSCLC accounts for approximately 81% of all lung cancers, and SCLC accounts for approximately 14% of all cases (1).

In this review, we will focus on treatment strategies and optimizing the management of brain metastases (BM) in patients with NSCLC.

## Clinical diagnosis of brain metastasis

**Figure 1:**
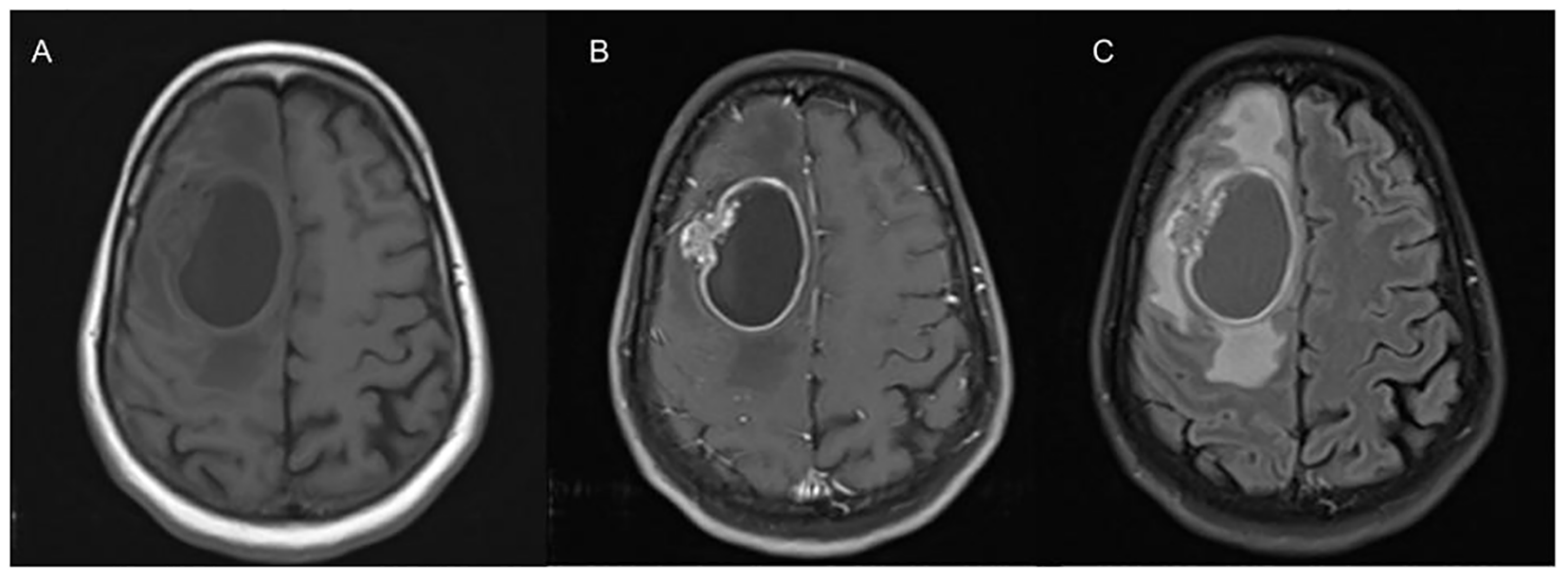
This is an axial view of T1 **(A)** revealing an ovoid-shaped hypodensity representing a cystic lesion in the right frontal lobe with perilesional edema with mass effect on contralateral hemisphere and posteriorly. On T1 post contrast imaging **(B)**, there is hyperintensity representing enhancement in the margins with heterogenous hyperintensity on the lateral wall appearing to invade brain parenchyma. FLAIR imaging **(C)** showing significant perilesional edema causing effacement of the sulci.

Early detection of BM is crucial in the management of NSCLC, hence the National Comprehensive Cancer Network (NCCN) guidelines recommend brain magnetic resonance imaging (MRI) with contrast with all clinical stage II disease or greater to identify BM in patients with symptoms, or occult BM (3). A contrast-enhanced Computed tomography (CT) brain is recommended for individuals with a contraindication to MRI (3). Contrast-enhanced MRI with T1-weighted images and T2-weighted fluid-attenuation inversion recovery (FLAIR) is most commonly used to identify BM, which provides information on size and morphological characteristics (4). Metastases typically appear iso- or hypointense on T1-weighted images, and variable in intensity on T2-weighted imaging (5). Presence of edema is identified using FLAIR sequencing and metastasis enhance on postcontrast imaging (5). The sensitivity of detecting BM with CT imaging is lower than MRI (5). An example of a patient with a BM in NSCLC is in **Figure 1**.

### Pattern of metastases

Metastases can manifest as solitary, multiple, or as leptomeningeal disease. Intracranial metastases typically exhibit enhancement post-contrast as they lack a blood-brain barrier (5). The enhancing patterns may present as either ring-enhancing lesions or solid nodules (6). In differential diagnoses, ring-enhancing lesions on MRI necessitate consideration of various pathologies such as gliomas, metastatic lesions, abscesses, multiple sclerosis lesions, and other less common etiologies (7). T2-hypointensity trends might help differentiate between different pathologies, for instance, a hypointense arc on T2 and heterogenous center is more indicative of a tumor, whereas multiple enhancing lesions are more suggestive of metastases (7). Nodular enhancing lesions can be attributed to various etiologies including hematogenous spread of infections or metastatic disease (5). Discriminating single ring-enhancing metastatic lesions from glioblastoma remains a challenge, as they often appear similar on standard MRI imaging (5). Integration of advanced imaging techniques and the incorporation of artificial intelligence can aid in the detection and classification of these tumors (4). Furthermore, assessing the total extracranial disease burden in such patients is imperative, for which imaging techniques such as positron emission tomography (PET/CT) or CT are employed.

## General principles of management of BM

A range of considerations influence the choice of treatment for BM in NSCLC, including factors such as BM size, number, location, associated symptoms, the presence or absence of actionable mutation, and the preferences of both patient and physician. Local therapies, such as surgery, and stereotactic radiosurgery (SRS) are typically recommended for symptomatic brain metastases, and in cases where patients have a limited number of BM.

According to guidelines from American Society of Clinical Oncology (ASCO)/American Society for Radiation Oncology (ASTRO), it is recommended that all patients with symptomatic BM should be offered local therapy. Specifically, surgery is preferred in patients with large or solitary tumors causing significant mass effect (8). Local therapies can be considered in patients who have a limited number of BM. There is no consensus on what accounts for limited brain metastasis, however, most studies define limited as less than 4 BM (9–13). In patients with multiple brain metastases, surgery is considered only when required for acute or impending symptomatology. The utilization of SRS has broadened in clinical practice to encompass patients with up to 10 metastases (14). However, despite this expansion, there is a lack of randomized data supporting its use in this context. The optimal selection criteria for patients with more than 4 BM remains contentious and is currently under investigation in ongoing research efforts.

## Treatment sequencing and individualized treatment approaches with shared decision-making

All patients with symptomatic brain metastases should be offered local therapy with surgery or radiation therapy in addition to systemic steroids to minimize swelling or vasogenic edema that may be present. Refer to sections covering their role for detailed discussion.

With the significant growth in treatment options and shifting landscape for patients with advanced NSCLC, it becomes increasingly important to have an open discussion with the patient regarding their current clinical and molecular status, findings, and how it influences their treatment options. At this time, more people are living with and surviving from lung cancer than ever before. Shared decision-making with the patient has become pivotal and the oncologist needs to guide patients to determine the best individualized treatment plan based on treatment side effects, functional status, goals of care, and patient expectations regarding quality of life. A multi-disciplinary approach and discussion between neurosurgery, medical oncology, radiation oncology, and neuro-oncology, along with the patient preferences is important to develop an individualized treatment plan.

## Surgery for BM and management of post-surgical cavities

Multiple advances in the field of neurosurgery have led to improvements in surgical techniques. Preoperative Functional MRIs along with high-resolution MRIs can identify high-risk structures and facilitate safer surgical planning (15). Minimally invasive craniotomy and keyhole approaches for tumor resection can achieve preservation of normal brain parenchyma without affecting the extent of tumor resection. Neuronavigation, which was first introduced in 1986 (16), has become an important tool in identifying the location of tumors and nearby structures. The use of intraoperative ultrasound, intraoperative mapping, and endoscope has also improved surgical techniques, making neurosurgery safer (15).

The role of surgery in patients with single BM in addition to WBRT was established in 1990 by Patchell et al. (17), as resection improved survival relative to WBRT alone. However, surgery alone was deemed suboptimal following a second randomized control trial by Patchell et al. (18), in which patients were assigned to either receive postoperative WBRT or observation. WBRT reduced rates of recurrence at both original sites (10% vs. 46%) or elsewhere in the brain (14% vs. 37%), however, no difference in overall survival was noted. Over time, there has been increasing use of adjuvant Stereotactic radiosurgery (SRS) to the surgical cavity as an alternative to WBRT in patients who have a single or limited number of BM (11, 19–23).

Mahajan et al. (24) recruited patients from a single center, who had complete resection of 1-3 BM with a maximum diameter of 4 cm. Patients were randomly assigned in a 1:1 ratio to either receive SRS within 30 days of surgery or observation. 12-month freedom from local recurrence was 43% in the observation group and 72% in the SRS group. Kocher et al. (12) in their study of adjuvant WBRT after radiosurgery or surgical resection of 1-3 cerebral metastases showed a reduced 2-year relapse rate at the initial site (27% vs 59%) or elsewhere (23% vs 42%), without significant difference in overall survival. These studies recapitulate the results of the original Patchell data, emphasizing the need for adjuvant radiotherapy to optimize control while underscoring the appropriateness of SRS as opposed to WBRT as an attractive strategy. Concern surrounding the neurocognitive effects (25–27) of WBRT fueled further interest in an approach that would spare unnecessary brain irradiation.

In the NCCTG N107C/CEC.3 randomized controlled phase 3 trial, 194 adult patients with one resected BM from 48 centers in the USA and Canada were randomly assigned to either postoperative SRS vs. WBRT (28). A battery of neuro-psychiatric tests were performed prospectively on the study population to evaluate a potential difference between the two radiotherapy approaches. The findings demonstrate that the cognitive deterioration-free survival was longer in patients assigned to the SRS group vs. the WBRT group (3.7 months vs. 3 months). Median overall survival (OS) was 12.2 months in the SRS group vs. 11.6 months in the WBRT group. These data serve to cement the notion that WBRT can be avoided without compromising survival and offer one of the best descriptions of neurocognitive comparisons of SRS and WBRT to date.

Adjuvant SRS has traditionally been delivered in a single fraction. However, the use of fractionated SRS (fSRS) has increased over time (for both resected and *in situ* lesions) with the thought that it may reduce the risk of radiation necrosis (RN) while maintaining local control (29), especially in larger targets. No prospective comparative data are available, but a recent cooperative group trial (Alliance A071801 - Clinical trial ID: NCT04114981) (30) randomized surgical cavity to single vs multi-fraction SRS will likely be instructive on the matter.

As described above, local failure rates at the surgical cavity approach are around 60% without the use of adjuvant radiotherapy (12), and the traditional approach utilizes RT following surgery. However, an alternative paradigm has emerged which offers SRS before surgical resection. A retrospective multi-institutional analysis suggested that preoperative SRS may reduce the risk of leptomeningeal recurrence, among other treatment-related conveniences (31). NRG Oncology is leading a randomized trial comparing pre- and post-operative SRS with a primary endpoint evaluating local tumor progression, adverse radiation effect, and nodular leptomeningeal recurrence. (NRG BN012, Clinical trial ID: NCT05438212) (32).

## Role of radiation in the management of unresected BM

The decision between surgical resection and stereotactic radiation (SRS) for patients with a single BM, is individualized, as there is no clear evidence demonstrating the superiority of one approach over the other. Treatment choices should be made on a case-by-case basis, considering the patient’s specific medical condition and preferences (33). Most data available is inclusive of trials that enroll all patients with BM and not just NSCLC.

SRS has long been used in patients who have a limited number of BM. There were several trials conducted to evaluate its utility alongside WBRT, or vice versa. In the randomized phase III RTOG 9508 trial, 333 patients with one to three BM were assigned to receive WBRT alone or WBRT with an SRS boost (13). This study demonstrated a survival benefit with the use of a boost in those with a single metastasis (median survival 6.5 versus 4.9 months, p = 0.0393) (13). There did not appear to be a benefit to the use of a boost with 2-3 lesions, and despite these results, routine use of radiosurgical boost following WBRT did not persist in routine practice.

Other studies asked the inverse question of whether routine use of WBRT had a benefit in addition to SRS for limited BM. In a phase III trial, JRSOG 99-1 (34), which randomly assigned 132 patients, with 4 or fewer BM to receive SRS with or without WBRT, the median survival was 7.5 months in the WBRT plus SRS group and 8 months in the SRS group alone (p = 0.42). However, the 12-month brain tumor recurrence rate was 46.8% for the SRS plus WBRT group versus 76.4% for the SRS alone group (p = <0.001). In the EORTC 22952-26001 study with a similar design (though local therapy could be either SRS or surgery) (12), 199 patients received SRS, out of which 100 patients were assigned to the observation group and 99 patients were assigned to the WBRT group. Overall survival was similar in both groups, however, WBRT reduced the 2-year relapse rate at the initial sites (19% vs 31%; p=0.04), and at new sites (33% vs 48%; p=0.023). Finally, Brown et al. (11) published the results of the N0574 trial in 2016, which randomized 213 patients with 1-3 BM to WBRT or observation following SRS. Consistent with prior results, WBRT improved distant brain control without improving overall survival. Less cognitive decline was seen at 3 and 12 months in the observation arm.

Taken together, these trials suggest that despite the improved intracranial control rates, WBRT can be omitted in those with a limited number of BM without compromising survival. This is largely due to the option of radiosurgical salvage of distant brain failures. This has been a welcome conclusion in the neuro-oncology world, given the above.

As WBRT remains an important treatment in those who aren’t good candidates for management with SRS alone, there has been significant interest in reducing the neurocognitive impacts. RTOG 0614, investigated the use of memantine, an NMDA receptor agonist, as a possible mitigator (35). In this randomized, double-blind, placebo-controlled trial, patients in the experimental arm had a significantly longer time to cognitive decline (hazard ratio 0.78 (CI 0.62 - 0.99; p=0.01), though the primary endpoint of the trial (delayed recall at 24 weeks) just missed statistical significance (53.8% versus 64.9%; p= 0.059) (35). Memantine is now routinely recommended alongside WBRT for 6 months based on these data.

Though it is incompletely understood, a key component of the neurocognitive toxicity and memory deficit may be a result of injury to hippocampal neural stem cells, marked by increased apoptosis and reduced neurogenesis (36). Efforts to protect the hippocampus aim to partially spare this effect. Intensity-modulated radiotherapy can be used to preferentially avoid the hippocampal stem cell compartment during WBRT (HA-WBRT) to this aim. A single-arm phase 2 trial of HA-WBRT using prespecified comparison with a historical control group without hippocampal avoidance (37). HA-WBRT was then tested in a phase III trial (NRG CC001), randomizing against patients receiving traditional WBRT. 518 patients were randomized and stratified by RPA class, and prior receipt of SRS/surgery or not (38). The primary endpoint was time to cognitive failure, and both arms received a dose of 30 Gy in 10 fractions. The trial was positive, with less deterioration in executive function (at 4 months) and learning/memory (at 6 months) in the HA-WBRT arm. Patient reported outcomes also favored the novel approach, and there was no difference in survival or intracranial progression.

These improvements in the delivery of WBRT have been incorporated into routine practice, though controversy remains regarding optimal scenarios for its use in up-front settings, compared with SRS. As noted earlier, the majority of trials enroll between one to four BM, but a well-defined upper limit that is appropriate for SRS remains elusive. A landmark publication from the Japanese detailed their prospective observational study data on the management of up to 10 BM in 1194 patients (14). All were managed with SRS, and those with a single lesion had better OS, but when grouped in 2-4 vs 5-10, there was no apparent difference. A more recent multi-institutional report of over 2000 patients similarly suggested no difference in survival between 2-4 BM and 5-15 BM cohorts - though the latter composed only 10% of the patients (39). It has now become common practice to offer SRS for properly selected patients with up to 10 (or more) lesions. Selection criteria should include age, performance status, size/location of tumors, systemic disease control, and availability of effective systemic therapy. There are clinical trials ongoing in space hoping to better define who benefits best from which approach, including most notably CCTG. CE.7 trial (Clinical trial ID: NCT03550391) (40), comparing HA-WBRT with SRS for patients with 5 or more lesions.

## Systemic therapies for brain metastases

The role of systemic therapy in treating NSCLC with brain metastasis is not well-defined due to limited trials focused solely on brain metastasis control. Most studies exclude patients with brain metastases. Systemic therapies include chemotherapy, immunotherapy, and targeted therapy. Per NCCN guidelines, systemic therapy alone may be considered for select patients with small, asymptomatic brain metastases, using agents with good CNS penetration (3). It’s reasonable to delay radiation therapy, though the impact on neurologic deficits or survival isn’t well-documented. Close MRI surveillance is recommended with the option to initiate radiation if needed while on systemic therapy along with radiation oncologist.

## Targeted therapies and their efficacy in brain metastases

Patients with driver mutations such as epidermal growth factor receptor (EGFR) mutation or Anaplastic lymphoma kinase (ALK) translocations with asymptomatic limited metastases may now be considered for systemic therapy as an early intervention.

Ongoing research is currently addressing the controversy surrounding our understanding of microenvironmental mechanisms within the brain and the potential breakdown of the blood-brain barrier (BBB) to improve the effectiveness of systemic therapies for the treatment of BMs (41). Current evidence suggests that EGFR tyrosine kinase inhibitor (TKI) therapy appears to be more effective than chemotherapy in controlling metastatic disease in the brain along with extracranial (EC) disease (41). Iuchi et al. (42), demonstrated that Gefitinib and Erlotinib, first-generation EGFR TKIs, have shown significant intracranial activity. A second-generation EGFR TKI, Afatinib has also demonstrated significant intracranial efficacy (43). The third-generation EGFR TKIs, AZD 3759, and Osimertinib have shown promising evidence of BBB penetration (44, 45). Additionally Osimertinib has the potential to exhibit sustained tumor regression and greater distribution into mouse brain tissue compared to Gefitinib, Rociletinib, or Afatinib (45). The FLAURA trial demonstrated that osimertinib had superior efficacy compared to standard EGFR-TKIs as first-line therapy for EGFR-positive (Exon 19 deletion or Exon 21 L858R mutation) NSCLC, for which 19% of patients, treated with Osimertinib, had intracranial (IC) metastases when enrolled (46). Patients who exhibit T790M-positive NSCLC have shown greater efficacy with osimertinib compared to Platinum/Pemetrexed, which includes CNS metastasis in a second-line setting (45). The FLAURA 2 trial demonstrated that among patients with brain metastases at baseline, the median progression-free survival was 24.9 months in patients who received Osimertinib with Pemetrexed plus Platinum-based chemotherapy versus 13.8 months in patients who received Osimertinib alone (47). Osimertinib is currently the preferred EGFR tyrosine kinase inhibitor for treating tumors with specific EGFR mutations, including the T790M mutation, NSCLC. Osimertinib CNS activity was evaluated using pooled data from two phase II studies including the AURA extension and AURA2. In this study, the primary outcome was the CNS objective response rate (ORR) assessed by a blinded independent central neuroradiology review (BICR). The confirmed CNS ORR was 54%. The median CNS duration of response was not reached within 1-15 months. At the 9-month mark, it was estimated that 75% of patients (with a 95% confidence interval of 53-88) remained in response (48). Other regimens that can be considered are pulsatile Erlotinib, Afatinib, daily Erlotinib, and Gefitinib. A *post hoc* analysis of the LUX-lung 3 as well as LUX-lung 6 demonstrated an increased progression-free survival (PFS) benefit (p= 0.0297) in patients with asymptomatic brain metastases treated with Afatinib at 8.2 months vs. standard chemotherapy at 5.4 months. From each group, roughly 33% of the patients had prior whole-brain radiation (43).

The incidence of BMs in Anaplastic lymphoma kinase (ALK) rearrangement NSCLC continues to be a problem. Targeted ALK rearrangement TKIs include Crizotinib, Ceritinib, Alectinib, Loratinib, and Brigatinib (41). The drugs developed after Crizotinib like Ceritinib, Alectinib, Brigatinib, and Lorlatinib induced a significant CNS response in those pretreated with Crizotinib (41). Phase 3 clinical trials looking at Brigatinib and Lorlatinib efficacy over Crizotinib, included 29% and 26% of patients, in the treatment arm, with IC metastases, respectively (49, 50).

There is also evidence that amivantamab is an EGFR-MET bispecific antibody. In the MARIPOSA study, a phase 3, randomized study evaluating the efficacy and safety of Amivantamab and lazertinib combination compared to osimertinib, showed that in a subgroup analysis for progression free survival in patients with BM, median PFS was 18.3 months (95% CI 16.6 - 23.7) for amivantamab-lazertinib group when compared to osimertinib, which was 13 months (95% CI 12.2 - 16.4) with hazard ratio of 0.69 (95% CI, 0.53 - 0.92) (51). In a single-arm phase 2 study, amivantamab and lazertinib with EGFR mutations, showed intracranial objective response rate of 40% for patients with BM and 23% for patients with leptomeningeal disease. In the MARIPOSA-2 study, in patients with EGFR mutated patients, amivantamab-lazertinib-chemotherapy, and amivanatmab-chemotherapy group had median intracranial PFS duration of 12.8 months and 12.5 months, when compared to chemotherapy alone, which was 8.3 months (52).

Identifying other clinically important mutations in NSCLC such as ROS1, MET, BRAF, NTRK and so on is important in identifying the most appropriate therapy for the patients. **Table 1** presents a summary of common targetable mutations in NSCLC and their CNS efficacy in NSCLC. The efficacy of oral TKI against RET rearragements is high. The efficacy of selpercatinib is mentioned in **Table 1**. Pralsetinib, another highly selective RET inhibitor, has shown efficacy with its ability to penetrate the blood brain barrier and have anti-tumoral effect. Pre-clinical studies has demonstrated activity in intracranial tumor models (53). The phase 1/2 ARROW study (54) demonstrated efficacy of pralsetinib in patients with NSCLC and brain metastases. A shrinkage of intracranial metastases was seen in all 9 patients with measurable brain metastases. 5 of 9 patients had an intracranial response and 3 had complete responses. Kaplan-Meier estimate of probability of ongoing intracranial response at 6 months was 80% and 53% at 12 months (54). Larotrectinib, a NTRK inhibitor, has also shown rapid and durable responses in patients with brain metastases, where the objective response rate has been 75% amongst use in all tumors (55). Entrectinib is also effective in intracranial disease in patients with NTRK mutation with objective response rates close to 60% with durable responses (12-month event free duration of response rate of 91%) (56). Repotrectinib has shown intracranial activity in ROS1 fusion-positive NSCLC (57). Of the patients with measurable metastases at baseline, intracranial response occurred in 8 of 9 (89%) who had not previously received a ROS1 TKI and in 5 of 13 (38%) who had previously received one ROS1 TKI and not received chemotherapy (57). There is not much data of efficacy of BRAF inhibitors in intracranial disease with NSCLC, however, there are some case reports which have shown benefit to vemurafenib for intracranial disease in NSCLC (58).

**Table 1:** Select targeted drugs with CNS efficacy with select studies demonstrating efficacy.

Drug	Targeted mutation	Type of study	Number of patients	Response	CNS PFS
Osimertinib	EGFR	Pooled analysis of 2 phase 2 studies (Goss et al.) (48)	50 with measurable CNS BM	ORR = 54% (95% CI 39 to 68)	Not reached (NR) at 11 months
		CNS response analysis of patients in FLAURA trial (Vansteenkiste et al.) (147)	61 patients analyzed in osimertinib arm	ORR=57%(95% CI, 44-70)	NR
Adagrasib	KRAS G12C	Phase 2 clinical trial (Janne et al.) (148)	42 patients out of 116 had BM (33 patients with radiologically evaluable BM)	ORR = 33% (95% CI 18-51.8)	5.4 months (95% CI 3.3 - 11.6)
Capmatinib	MET Exon 14 skipping mutation	Phase 2 clinical trial (Wolf et al.) (149)	14 of 97 with MET exon 14 skipping mutation (13 with evaluable BM)	7 of 13 patients with intracranial response and 4 patients with complete response	NA
Tepotinib	MET Exon 14 skipping mutation	Phase 2 clinical trial: *ad hoc* retrospective analysis to evaluate intracranial activity (Le et al.) (150)	23 of 152 patients with BM. 15 with evaluable BM and 7 measurable target lesions for response evaluation	Intracranial disease control in 13 patients. 5 of 7 measurable disease had partial response	NA
Selpercaptinib	RET fusion	Phase 1/2 clinical trial (Subbiah et al.) (151)	80 of 531 patients with BM. 22 patients with measurable BM.	ORR = 82% (95% CI 60-95). 23% patients with CR, 59% patients with PR. Intracranial disease control = 100%.	13.7 months (11.9 - not estimable)
Brigatinib	ALK rearrangement	Phase 3 clinical trial (Camidge et al.) (49)	90 of 275 patients with BM. 39 with measurable BM. 18 in the Brigatinib arm.	Overall ORR = 83% (95% CI, 59 - 96)	NA
		Phase 2 clinical trial (Kim et al.) (152)	153 of 222 patients with BM. 44 with measurable BM.	ORR = 42% (11 of 26 patients) in arm A (90 mg dose) and 67% (12 of 18 patients) in arm B (180 mg dose)	15.6 months (95% CI, 7.3 to 15.7) in arm A and 12.8 months (11.0 to not reached) in arm B
Lorlatinib	ALK rearrangement	Phase 3 clinical trial: *post hoc* analysis of intracranial efficacy (Solomon et al.) (50)	38 of 149 patients in the lorlatinib arm had BM.	ORR = 66%; Complete CNS response = 23/38 (61%);	NA
Alectinib	ALK rearrangement	Phase 3 clinical trial (Peters et.al.) (153)	64 of 152 patients in alectinib arm with BM. 21 patients with measurable disease.	CNS response= 17/21 patients. 8 patients with CR. Median duration of intracranial response = 17.3 months (95% CI, 14.8 to not estimable).	NA
		Pooled analysis from data from 2 phase 2 trials (Gandhi et al.) (154)	Measurable BM in 50 patients with RECIST criteria and 43 by RANO-HGG criteria.	CNS objective response =64%(95% CI, 49.2 - 77.1)(RECIST) and 53.5% (95% CI, 37.7-68.8) (RANO-HGG)	10.8 months with RESICT and 11.1 months with RANO-HGG
Ceritinib	ALK rearrangement	Phase 1 trial (Kim et al.) (155)	94 of 246 patients with BM. 36 with measurable BM based on RECIST 1.1.	Intracranial disease control rate=62·5% (5/8; 95% CI 24·5–91·5) in ALKi-naïve patients and 60·7% (17/28; 95% CI 40·6–78·5) in ALKi-pretreated patients. Duration of response= 8·2 months (95% CI 5·6–NE) in ALKi-naïve and 11.1 months (95% CI 2·8–NE) in ALKi-pretreated patients	NA
Entrectinib	ROS1 mutation	Updated analysis of 3 phase 1 or 2 trials. (Dziadziuszko et al.) (156)	46 patients with BM, and 24 patients with measurable BM.	Intracranial ORR in all patients with baseline CNS = 52.2% (n = 24; 95% CI, 37.0 to 67.1). intracranial ORR for patients with measurable disease was 79.2% (n = 19, 95% CI, 57.9 to 92.9	8.3 months (6.4 to 15.7) in all (measurable and unmeasurable). 12 months (6.2 - 19.3) in patients with measurable disease.

NA, not available.

The combination of RT and TKIs is promising and aims to optimize the effect of SRS, WBRT, or stereotactic radiotherapy (SRT). A retrospective study demonstrated that patients with exon 21 mutation treated with WBRT and TKI had significantly higher OS and PFS than TKI alone (59). Another possible option being investigated is the combination of immunotherapy and RT, with some recent retrospective studies looking at the SRS before and concurrently with checkpoint inhibitors (41). Pseudo-progression is known to occur in these combined modalities and should be considered when evaluating response to treatment. Further investigation in this area, through clinical trials, would assist in the timing, dosing of treatments, and appropriate imaging modalities.

Antibody-drug conjugates (ADCs) have become other targeted options that can be considered in certain patients with BM. In patients who have human epidermal growth factor receptor 2 - mutant (HER2 mutant) metastatic NSCLC, the use of trastuzumab deruxtecan has shown efficacy and safety in management of BM. In a pooled analysis from DESTINY-Lung01 (60) and DESTINY-Lung02 (61) study showed that patients with baseline BM showed intracranial efficacy with some complete responses (62). Patritumab deruxtecan is a humanized monoclonal antibody to HER3 attached to a topoisomerase inhibitor payload (63). In the phase 2 HERTHENA-Lung01 trial (63), patritumab deruxtecan was evaluated in EGFR-mutated NSCLC previously treated with EGFR TKI and platinum-based chemotherapy. In analysis of 30 patients with BM without prior radiation therapy, the CNS objective response rate was 33.3% (95% CI, 17.3 - 52.8) with 9 complete responses and one partial response. Median duration of intracranial response was 8.4 months.

It is crucial to consider that metastatic disease in the brain has the potential to exhibit genomic alterations distinct from those observed at the primary tumor site (64–66).This would imply that targeted therapies that may be effective outside the brain can fail to impact BM lesions (41). In cases where no actionable mutations were initially detected at the primary site, performing a biopsy of the brain metastasis lesion or identifying mutations through circulating or liquid genetic sequencing may uncover new actionable mutations that can be leveraged to explore additional treatment options (41). Further evaluation and clinical trials are needed to better understand and determine the best treatment options for patients with NSCLC BMs without mutation.

## Chemotherapy regimens and response rates

The approach of initially treating asymptomatic brain metastases with systemic therapy instead of radiation therapy aims to control both systemic disease and brain metastases simultaneously. However, it has been historically challenging to treat brain metastases with chemotherapy due to the limitations posed by the blood-brain barrier, which restricts the passage of many drugs into the brain. This limitation has led to the exploration of targeted therapies and other treatments with better central nervous system penetration for managing brain metastases in patients with metastatic lung cancer (67). A randomized pilot trial randomized 48 patients to either chemotherapy (Gemcitabine and Vinorelbine) followed by WBRT vs WBRT followed by chemotherapy (68). The study showed there was no significant difference in response rate or survival between groups. Franciosi et al. (69) in a prospective study of previously untreated patients with NSCLC treated with cisplatin and etoposide showed a complete response (CR) in 7% and CR or partial response (PR) in 30% of patients. The median survival was found to be 32 weeks. Another study looked at the use of a standard of care regimen for NSCLC, paclitaxel-cisplatin with the addition of either vinorelbine or gemcitabine, as front-line therapy in 26 chemotherapy-naive patients (70) which found that there was an intracranial response rate in 38% of the patients.

## Immunotherapies and their applications in BM

Immunotherapy drugs are classes of drugs that bind to programmed cell death-1 (PD-1) or programmed death ligand 1 (PD-L1). Although data is limited, both can be considered for systemic treatment in individuals with NSCLC and asymptomatic BM.

Hellmann et al. (71) evaluated the efficacy of Nivolumab and Ipilimumab as a frontline treatment for advanced NSCLC in the CheckMate 227 study. This trial showed that irrespective of the PD-L1 expression, the dual immunotherapy (IO) was superior with a median OS of 17.1 months (95% CI, 15.2 to 19.9) compared to 13.9 months with chemotherapy. Among the participants in the trial, 81 patients (10.2%) had CNS metastasis, and those receiving dual immunotherapy had a favorable median OS of 16.8 months with HR of 0.68 (0.41-1.11).

In a non-randomized, open-label, phase 2 trial conducted by Goldberg et al. (72), the role of Pembrolizumab was evaluated in patients with NSCLC who had untreated brain metastases and PD-L1 expression. The trial found that 6 out of 18 patients enrolled (33%) showed a positive response to Pembrolizumab treatment. This suggests a potential benefit of Pembrolizumab in this specific patient population. The CNS effect of Pembrolizumab in the phase 3 KEYNOTE-024 trial for patients with PD-L1 ≥50% was favorable (73). There were 18 participants (11.7%) with brain metastasis and the PFS rate HR was 0.55 with a CI of 0.20-1.56.

Gauvain et al. (74) in their retrospective multicenter study on patients with NSCLC and BM treated with Nivolumab, the primary endpoint of intracerebral objective response rate according to the RECIST criteria found only modest benefit with a 9% objective response rate in those who had pretreated BM vs. 11% in active BM.

The OAK trial compared atezolizumab to docetaxel in previously treated NSCLC which showed that in patients with CNS metastases, the median overall survival was 20.1 months in atezolizumab arm, when compared to 11.9 months in docetaxel arm with a hazard ratio of 0.54 (95% CI, 0.31-0.94), favoring the atezolizumab arm (75). The use of Durvalumab consolidation after chemoradiotherapy in stage III NSCLC has been associated with lower incidence of development of brain metastasis with a rate of 5.5% in the Durvalumab arm versus 11% in placebo arm (76). The EMPOWER-Lung study showed the efficacy of cemiplimab in patients with PD-L1 of at least 50% (77). Data from *post-hoc* analysis at a median follow up of 33 months among patients with clinically stable BM at randomization, cemiplimab prolonged median OS (NR vs 20.7 months) and median PFS (12.5 months vs 5.3 months) when compared to chemotherapy (78).

A meta-analysis performed by Chu et al. (79), 3160 participants with NSCLC and BM showed that patients treated with immunotherapy were associated with a longer PFS and a longer OS when compared to immunotherapy-naive patients. No obvious difference in PFS and OS was noted among different types of immune checkpoint inhibitors. The combination of immune check point inhibitors and anti-angiogenic agents has shown good anti-tumoral activity and tolerable safety profile and may have synergistic anti-tumor effect in NSCLC BM (80). It has been noted that antiangiogenic drugs may increase infiltration of T cells in BM after inhibiting VEGF (81). In a *post hoc* analysis of the IMpower 150 trial, lower rate of new BMs were noted in atezolizumab plus bevacizumab plus chemotherapy group when compared to atezolizumab plus chemotherapy group, giving a suggestion that combination of immunotherapy with VEGF inhibitors may have the potential of delaying occurrence of BM (82).

Combination with radiotherapy and immunotherapy may cause synergy through the release of danger associated molecular patterns (DAMPs) and cytokines (83). There are several retrospective studies that have highlighted the benefit of immunotherapy and radiotherapy (83). The phase I/II trial (NCT02696993) is currently recruiting to assess the side effects and efficacy of Nivolumab with SRS or WBRT with or without Ipilimumab. Safety data from phase I portion of concurrent nivolumab and ipilimumab with SRS has been reported and has been reported safe and has encouraging durable intracranial response (84).

## Impact of BM and treatment on neurocognitive function and rehabilitation strategies

Neurocognitive Function (NCF) impairment is noted to be extremely common in those with brain tumors, with one study finding 90% of its participants having some form of NCF deficits at baseline including fine motor control, executive function, and memory (85). The neurocognitive decline observed in individuals with NSCLC and BM appears to have a multifactorial pathology. Contributing factors include tumor invasion into the brain, medications used for symptomatic management (such as steroids), systemic chemotherapy, and WBRT (86).

The strategies for reducing the effects of WBRT are discussed in the section above.

## Leptomeningeal metastases

Leptomeningeal metastases, also known as leptomeningeal carcinomatosis, is an uncommon but grave complication of advanced lung cancer. Leptomeningeal metastases occur in 3-5% with NSCLC (87). Common symptoms of leptomeningeal metastasis include headaches, visual disturbances, cranial nerve deficits, seizures, and cauda equina syndrome among others (88). The gold standard for diagnosis of leptomeningeal disease is through cerebrospinal fluid (CSF) cytology, however, repeated lumbar punctures might be required for increasing sensitivity of CSF cytology (87). False negatives may still exist despite repeated examinations (89), and thus many times a clinical diagnosis needs to be made based on clinical and imaging findings. If CSF cytology remains negative for malignant cells, analysis of CSF protein is informative and is usually higher in patients with leptomeningeal disease (88). MRI is instrumental in diagnosing leptomeningeal carcinomatosis, necessitating comprehensive imaging of the entire neural axis, encompassing both the brain and spine (90). Leptomeningeal enhancement, which may present as either diffuse or focal enhancement due to tumor nodules can be noted on MRI imaging (90). Furthermore, there is growing evidence that use of cell-free DNA (cfDNA) from CSF could improve sensitivity and accuracy for diagnosing leptomeningeal metastases (91–94). These tests are currently only available at limited number of tertiary cancer centers, and as per the Americal Society of Clinical Oncology/Society for Neuro-Oncology consensus review, these techniques require validation and regulatory certifications before they can routinely be incorporated in clinical practice (95). NCCN guidelines (Central Nervous System Cancers, version 1.2023) stratify patients with leptomeningeal metastases into “good risk” [Karnofsky performance status (KPS) > 60, no major neurological deficits, minimal systemic disease, and reasonable treatment options] and “poor risk” [KPS <60, multiple neurological deficits, extensive systemic disease with few treatment options, bulky CNS disease and encephalopathy] (96). For patients with poor risk disease, palliative or best supportive care is considered, with consideration given to involved field radiation therapy (IFRT) for palliation of symptoms. In patients with good risk disease, systemic therapy, intra-CSF therapy or radiation therapy can be considered (96). When radiation is considered, IFRT in the form of Whole brain radiation therapy (WBRT) and/or focal spine RT is used (97). When treating patients with systemic treatment, systemic therapies with good CNS penetration should be selected. In patients with targetable mutations such as EGFR mutation, treatment with targeted therapies such as Osimertinib and ‘pulsatile’ high dose Erlotinib have shown responses in CNS disease and leptomeningeal metastases (98–100). In a single-arm phase 1/2 clinical trial involving patients with EGFR-mutant NSCLC and leptomeningeal metastases who had previously failed tyrosine kinase inhibitors, the administration of intrathecal (IT) Pemetrexed with dexamethasone demonstrated a noteworthy clinical response rate of 84.6%. Additionally, the trial reported a median overall survival rate of 9 months among participants (101). These findings suggest that such a treatment approach could be a viable consideration for patients with similar characteristics. Other chemotherapeutic drugs used for intrathecal chemotherapy in patients without targetable mutations and with good performance status include IT Methotrexate, Cytarabine, and Thiotepa (102).

## Follow-up and surveillance strategies for brain metastases

### Imaging modalities for surveillance

The gold standard for brain tumor imaging is MRI due to its excellent tumor delineation and high tissue anatomy resolution. However, it’s sensitive to movement, not suitable for patients with certain implants, and can be problematic for claustrophobic individuals. CT with and without contrast is an alternative for these cases, but it has a lower resolution and may not be ideal for those with renal issues. Specialized tests like MR spectroscopy (metabolite assessment) and MR perfusion (cerebral blood flow measurement) can help distinguish true progression from pseudoprogression, guide response monitoring, or assist in biopsy target selection. However, they may have limitations when tumors are near bone, vessels, or air spaces.

### Monitoring treatment response and disease progression

In the setting of surgical resection of a brain tumor it is recommended to obtain imaging, preferably with MRI with contrast, 48-72 hours post-surgery to avoid surgery-related enhancement which can frequently mimic a residual tumor. Imaging post-surgery has been found to help guide subsequent treatment (103). The recommendations thereafter are to obtain a Brain MRI every 2-3 months for 1-2 years and then every 4-6 months indefinitely if there are no further changes or concerns (104, 105).

If there are any changes in the patient’s signs or symptoms, repeating imaging is appropriate to assess their condition.

The Response Assessment in Neuro-Oncology Brain Metastases (RANO-BM) group, is an international, multidisciplinary effort to develop progression and response criteria for brain metastases, which is commonly used to assess response in clinical trials and practice (106). The best overall CNS response is defined as complete response (disappearance of all CNS target lesions sustained for 4 weeks with no use of steroids), partial response (30% decrease in the sum of longest diameter of lesion), progressive disease (20% or more increase in the sum of longest diameter of lesion, or unequivocal presence of new lesion), or stable disease (Neither sufficient shrinkage or increase to qualify for progressive disease or partial response) (106).

## Management of recurrent or progressive brain metastases and radiation necrosis

Recurrent or progressive brain metastases are metastases that recur in either the original site or non-original site after initial therapy. When dealing with recurrent or progressive brain metastases, it’s advisable to involve a multidisciplinary team to create an individualized treatment plan. This approach is recommended because there is currently insufficient data to definitively compare the overall benefits of one therapy over another, leading to a lack of definitive treatment recommendations. An individualized plan takes into account the unique characteristics and needs of each patient to provide the best possible care. These factors are similar to those mentioned above when discussing WBRT vs SRS. Many of these clinical factors may have changed since the initial decision regarding the management of the BM.

Depending on the scenario and the nature of the recurrence, options include re-irradiation (if local failure), either WBRT or SRS, surgical excision, or systemic therapy. Limited number of distant brain progressive lesions are typically managed with continued SRS, unless acutely symptomatic. Repeat WBRT is generally avoided given increased risk of toxicity, unless other options are infeasible,and the patient remains reasonably well controlled, systemically. Progression within a treated lesion can be more challenging, given the differential of radiation necrosis (RN), which is another important complication of radiation therapy. The exact incidence of RN is not known, but ranges between 0 to 30% (107). Differentiating between RN and tumor progression is important but can be challenging to delineate (107). Pathological assessment of tissue is the gold standard for diagnosis, however, is not always feasible due to the potential complications from surgery required to obtain tissue (108). On conventional MRI, RN usually appears as a ring-enhancing lesion on T1-weighted imaging, with surrounding T2/FLAIR signal, which represents vasogenic edema, which is non-specific and may also be seen in setting of tumor recurrence (108). Perfusion weighted MRI can help distinguish between recurrence of tumors from RN (109). It is theorized that as viable tumor has intact vasculature which leads to higher perfusion and increased relative cerebral blood volume (rCBV), which is not the case in RN, in which case the rCBV will be low (108, 109). Other imaging techniques such as magnetic resonance spectroscopy (MRS) and fluorodexoyglucose (FDG) and amino acid positron-emission tomography (PET) can be considered to differentiate between RN and tumor progression, however the Response Assessment in Neuro-Oncology Brain Metastasis group suggests that at this time current literature is ‘insufficiently robust’ to recommend any one particular modality routinely (107). In symptomatic patients with RN, the initial treatment is usually with a glucocorticoid, favorably dexamethasone, which provides symptomatic relief by improvement in cerebral edema. In patients who do not achieve symptomatic relief, non-surgical options include bevacizumab and laser interstitial thermal therapy (LITT) (110, 111). Two small randomized trials and retrospective case series have indicated bevacizumab may be useful in certain cases. In a double-blinded clinical trial, 14 patients with RN were randomly assigned to receive bevacizumab (7.5 mg/kg every 3 weeks for 4 doses) or saline-placebo which noted that all patients who received bevacizumab had improvement in neurological symptoms or imaging findings (112). Another open-label trial compared bevacizumab (5 mg/kg every 2 weeks for 4 doses) with glucocorticoids in patients with nasopharyngeal carcinomas with 112 patients showed that there was increased rates of radiological response (65.5 versus 31.5%) and clinical improvement (62.1 versus 42.6%) at 60 days (113). LITT is also a potential option for patients with RN which is effective in local control and symptom management (110, 114). Finally surgical resection of the necrotic tissue may sometimes be required, which can achieve palliative benefit and reduce mass effect.

The available evidence in management of recurrent BM is predominantly class III, comprising case series, concerning retreatment of brain metastasis with WBRT, SRS, or surgery. Notably, three distinct case series have investigated the use of WBRT for recurrent BM following WBRT as the initial treatment. The average re-irradiation dose of 20 to 25 Gy was delivered in multiple fractions, with a post-re-radiation median survival ranging from 4 to 5 months (115–117). Conversely, treatment with SRS subsequent to WBRT as an initial intervention yielded median survival from 4.5 to 19 months across different case series (118–120). When SRS was administered following SRS as the initial treatment, the median survival extended to 22.4 months, with 72% of patients achieving complete response, while the remaining 27% exhibited partial response or stable disease (121). Furthermore, the outcomes associated with surgery after SRS as the primary treatment demonstrated a median survival of 11 months, with a time to relapse within the brain of 5 months (122). These retrospective data contain significant bias, however, as those selected for one modality or another carry with them confounding variables that contribute to outcome. One factor that has emerged to potentially aid in decision making between WBRT and SRS is brain metastasis velocity (BMV). BMV is defined as the ratio of new lesions developing over time, and increasing value is associated with worse outcome (123). There is an ongoing trial evaluating SRS vs WRBT in patients with high BMV (NRG BN009, clinical trial ID: NCT04588246) (124).

## Multidisciplinary approaches and care in brain metastases

### Integrating supportive care services for comprehensive patient management

Supportive care services such as palliative care and integrative medicine have shown to be extremely valuable to patients with cancer as well as their loved ones by helping in areas such as quality of life, depression, anxiety, health care utilization, and even survival thus improving their overall experience (125). In 2009 a randomized controlled trial, involving 312 patients with advanced cancer (35% with lung cancer) provided a psychoeducation intervention consisting of four weekly educational sessions and monthly follow-up sessions. It showed an improvement in quality of life and a reduction in depressed mood (126). The following year a single institution randomized controlled trial evaluated early referrals to palliative care with standard oncology care versus standard oncology care alone. There were 151 patients with newly diagnosed metastatic non-small cell lung cancer. It was found that there were fewer depressive symptoms in the palliative care group (6% vs. 38%, respectively; p = .01). Also, although fewer patients in the early palliative care group received aggressive end-of-life care compared with the standard care group, the median survival was longer among patients receiving palliative care (11.6 vs. 8.9 months; p = .02) (127). Certain symptoms can serve as indicators to healthcare providers that a patient with advanced illness is approaching the end of life. These symptoms may include fatigue and increased confusion, along with headaches, blurry vision, swallowing or speaking difficulties, focal weakness, or seizures. These symptoms can lead to reduced oral intake, malnutrition, dehydration, and aspiration, which can cause dyspnea or pneumonia. Initial treatment with steroids can help alleviate many of these symptoms. For further management, oral medications may be used to prevent seizures and manage anxiety. When patients can no longer take pills, they can switch to liquid lorazepam. Nausea can be addressed with medications like disintegrating ondansetron, metoclopramide, prochlorperazine, and lorazepam. Open communication with caregivers is crucial to prepare them for expected symptoms and provide information on available resources, including hospice care (128).

## Prognostic factors and treatment decision-making in brain metastases

### Predictive factors for treatment response and survival outcomes

The overall survival of patients diagnosed with BMs remains poor but has improved over the last couple of decades (129). The prognosis may depend on a multitude of different factors such as age, performance status, time from diagnosis, disease activity, as well as the disease location and extension into the intra or extracranial space (41).

The Radiation Therapy Oncology Group (RTOG) developed a prognostication tool, recursive partitioning analysis (RPA) based on Karnofsky performance status (KPS), primary tumor status, presence of extracranial disease, and age which divides patients into three classes (130). However, this scale does not take into account the pathological and molecular features of the tumor into account while determining prognostication. More recently, a diagnosis specific graded prognostic assessment (GPA) is being used to determine prognostication based on types of cancers, histology and molecular factors such as EGFR, ALK and PD-L1 status (131). An online calculator is available.

More recently, with the ongoing development of advanced targeted therapies, we must identify predictive and prognostic factors to help guide treatment. This information has the potential to not only aid in preventing BMs but may also provide further insight into specific treatment options, which may better act upon and treat the disease that has spread to the brain.

In NSCLC, several predictive and prognostic biomarkers have emerged, and testing should be performed on all locally advanced to stage IV NSCLC patients (132). Predictive biomarkers to be tested for, as per the NCCN guidelines (3), are listed in **Table 2**.

**Table 2:** Predictive Biomarkers.

	Predictive
Molecular Biomarkers (somatic genomic alterations)	ALK rearrangements
	BRAF p.V600E point mutations
	EGFR mutations
	ERBB2(HER2)^*^
	KRAS^†^ mutations
	METex14^‡^ skipping mutations
	NTRK1/2/3^§^ gene fusions
	RET^¶^ rearrangements
	ROS proto-oncogene 1
Immune Biomarkers	PD-L1

*ERBB2(HER2) = v-erb-b2 avian erythroblastic leukemia viral oncogene homolog 2 (human epidermal growth factor receptor 2).

^†^KRAS = Kirsten Rat Sarcoma virus.

^‡^METex14 = mesenchymal-epithelial transition factor exon 14.

^§^NTK1/2/3 = neurotrophic tyrosine receptor kinase 1/2/3.

^¶^RET = rearranged during transfection.

The risk of acquiring BMs in the setting of particular biomarkers continues to be evaluated. Li et al. (133) performed a meta-analysis of 22 studies, incorporating 8,152 patients, to evaluate the correlation between EGFR status with the incidence of BMs in NSCLC and concluded that EGFR mutations are associated with a significantly higher incidence of BMs. This increased incidence is attributed to the downstream effects of EGFR on activated MET through the mitogen-activated protein kinases (MAPK) pathway and the STAT3 pathway which increases the expression of IL6 (133). Similarly, harboring an ALK mutation results in the production of a fusion protein which ultimately activates a signal transduction cascade, cell proliferation, inhibition of apoptosis, and eventually the stimulation of tumor growth; therefore patients with ALK-positive NSCLC are at higher risk of developing BMs (134). In fact, a study looking at the comparison of ALK-positive versus EGFR-positive patients showed that ALK-positive tumors exhibited greater metastatic ability, particularly when it came to brain metastases (135).

The presence of KRAS mutations is a poor prognostic marker of survival for patients with NSCLC, compared to those who do not harbor the KRAS mutations, independent of therapy, and have shown to have increased rates of BM development (136, 137). ROS1-positive NSCLC has lower rates of brain mets compared to EGFR/ALK mutations (138). Another study demonstrated that there was no statistically significant difference in BM development based on ROS1, ALK, EGFR, KRAS, or BRAF mutation status (139). Although the discovery of these biomarkers has led to nuanced targeted agents, the impact of these alterations (EGFR, ALK, KRAS, ROS1, RET and others) on BM development and the explanation behind this association still need to be fully elucidated (137).

Identifying patients with driver-oncogene mutations continues to drive treatment options as next-generation ALK inhibitors and TKIs have been associated with significant intracranial response rates and are effective in BMs (140). Specifically, third-generation EGFR-TKI osimertinib proved to have an intracranial response rate of over 80% while ALK inhibitors including Alectinib, Lorlatinib, and Brigatinib had an intracranial response rate of over 65% (140).

Similarly, the oncology community wanted to know the implications of immune biomarkers and their impact on the development of BM. Lee et al. (141) investigated the association of PD-L1 expression and BM in NSCLC patients and found that tumors exhibiting positive PD-L1 expression had a higher frequency and risk of developing BM. Unfortunately, BM has shown variable response rates to anti-PD-L1 therapies, possibly due to PD-L1 discordance between primary and BM (142). Additional studies are needed to better understand the mechanism behind this.

## Clinical trials and emerging treatments for brain metastases

### Overview of ongoing clinical trials in brain metastases

Clinical trials play an important role in the treatment of metastatic diseases and allow physicians to find new ways to improve quality of life and prolong survival. The US Food and Drug Administration (FDA) has made recommendations to include patients with brain metastases in cancer clinical trials (143). It is recommended to include patients with BM in a separate subgroup within the trial. Patients with stable or treated BM should be included in clinical trials unless there is a strong rationale to exclude them. Patients with active BM or leptomeningeal disease should not be automatically exluded from trials and should be included in clinical trials if there is a strong likelihood of CNS activity of any drug being investigated, unless known CNS toxicities exist or there is an immediate need of CNS specific treatment (143). In early clinical development for drugs with potential to increase risk of bleeding, patients with hemorrhagic BM or receiving anticoagulation should be excluded. Also with drugs with a potential to lower seizure threshold, patients should be carefully assessed and exclusion of these patients till safety data is available may be important (143). Currently, ongoing trials are looking into combined targeted therapies to enhance the efficacy of RT treatments to BMs. Other studies focus on understanding current targeted therapies and their ability to pervade the BBB to reach CNS tumors.

One important aspect to recognize is that NSCLC BMs may result in a common lineage and continue to evolve to acquire genomic alterations not found at the primary site (144). For instance, gene mutations were evaluated across BMs in breast, lung, and melanoma for which a new PIK3CA gene mutation has been identified, suggesting that PIK3CA may play a role in the metastatic process in the brain which could improve targeted treatment modalities (144). The ongoing challenge remains to be the difficulty of drug permeation across the BBB and the study of the pharmacokinetics of drugs in IC metastasis.

### Investigational therapies and their mechanisms

There are hundreds of clinical trials currently recruiting patients to look more closely into the mechanism of metastatic brain disease in NSCLC and how it can be effectively treated to impact PFS and OS. Recently a variety of treatments such as chemotherapy, EGFR TKIs, Temozolomide, Nitroglycerin, Endostar, Enzastaurin, and Veliparib have been added to RT to determine impact on newly diagnosed BMs (145). A portion of these studies have already been completed and some have required further investigation.

To provide a few examples, the oncology community has expressed interest in exploring the combination of icotinib, a first-generation EGFR TKI, with WBRT for the treatment of EGFR-mutated NSCLC that has spread to the brain (146). There has also been continued interest in the proven efficacy of other EGFR TKIs, such as Osimertinib and Almonertinib, in combination with RT treatments such as WBRT and SRS. Also under investigation are other treatments proven to help in other primary brain tumors, such as Temozolomide, in combination with RT for NSCLC BMs (145).

### Promising results and potential future treatments

Due to the recent and rapid development of targeted therapies in NSCLC and BMs this has been a focus for clinical trials. However, there are still a significant number of patients who are negative for both the EGFR/ALK mutations (145), and therefore future clinical trials would benefit from optimizing therapy options for patients with non-targetable NSCLC BMs.

Given the specificity of the biology of BMs, novel clinical trials that specifically target the microenvironment have been and continue to be evaluated. Combining specific treatment modalities and where to sequence them will prove to be immensely important as current trials navigate between the best combinations of chemo/immunotherapy, targeted therapy, surgery, and/or radiation treatment options.
